# Small Intestinal Neurofibroma With Atypical 17q11.2 Microdeletions: A Rare Cause of Abdominal Distension

**DOI:** 10.1002/jgh3.70138

**Published:** 2025-04-12

**Authors:** Yating Wang, Chunwei He, Dedong Ma, Leiqi Xu

**Affiliations:** ^1^ Department of Pulmonary and Critical Care Medicine Shandong University Qilu Hospital Jinan Shandong China; ^2^ Department of Gastroenterology Shandong University Qilu Hospital Jinan Shandong China

**Keywords:** 17q11.2 microdeletion, Neurofibromatosis Type 1, small intestinal neurofibroma

## Abstract

**Background:**

Neurofibromatosis Type 1 (NF1) is a rare autosomal dominant disorder caused by mutations or deletions in the NF1 gene, with approximately 5% to 11% of cases specifically attributed to the 17q11.2 microdeletion. While cutaneous manifestations are common, gastrointestinal involvement occurs in 10%‐25% of cases, with symptomatic presentations being exceptionally rare. This report describes an unusual case of NF1 presenting with small intestinal neurofibroma, emphasizing diagnostic challenges and management strategies.

**Case Presentation:**

A 22‐year‐old male with a 1‐year history of recurrent abdominal distension was admitted. Physical examination revealed pathognomonic features of NF1, including axillary freckling and café‐au‐lait macules. Laboratory tests demonstrated anemia and hypoalbuminemia. Imaging and enteroscopy identified a stenotic ileal lesion with mesenteric lymphadenopathy. Initial biopsy suggested neurofibroma, confirmed by whole‐exome sequencing revealing a 17q11.2 microdeletion involving the NF1 gene. Following palliative ileostomy, definitive surgical resection achieved complete remission. Histopathology confirmed small intestinal neurofibroma in NF1.

**Conclusion:**

This case underscores that gastrointestinal neurofibromas, though uncommon, should be considered in NF1 patients with persistent abdominal symptoms. A combination of clinical assessment, imaging, endoscopy, and genetic testing is essential for accurate diagnosis. Surgical intervention remains the definitive treatment for symptomatic lesions. The report expands the phenotypic spectrum of NF1 and highlights the importance of multidisciplinary management in rare gastrointestinal manifestations.

A 22‐year‐old man with a complaint of recurrent abdominal distension for 1 year was admitted to our hospital. He was diagnosed as “ulcerative colitis” by colonoscopy without specific medication. He has no family history. Physical examination revealed freckling at axillary (Figure 1A) and inguinal regions and diffused café‐au‐lait macules on skin (Figure 1B). Laboratory tests showed anemia (hemoglobin 76.0 g/L), fecal occult blood, and hypoalbuminemia (albumin 30.4 g/L).

**FIGURE 1:**
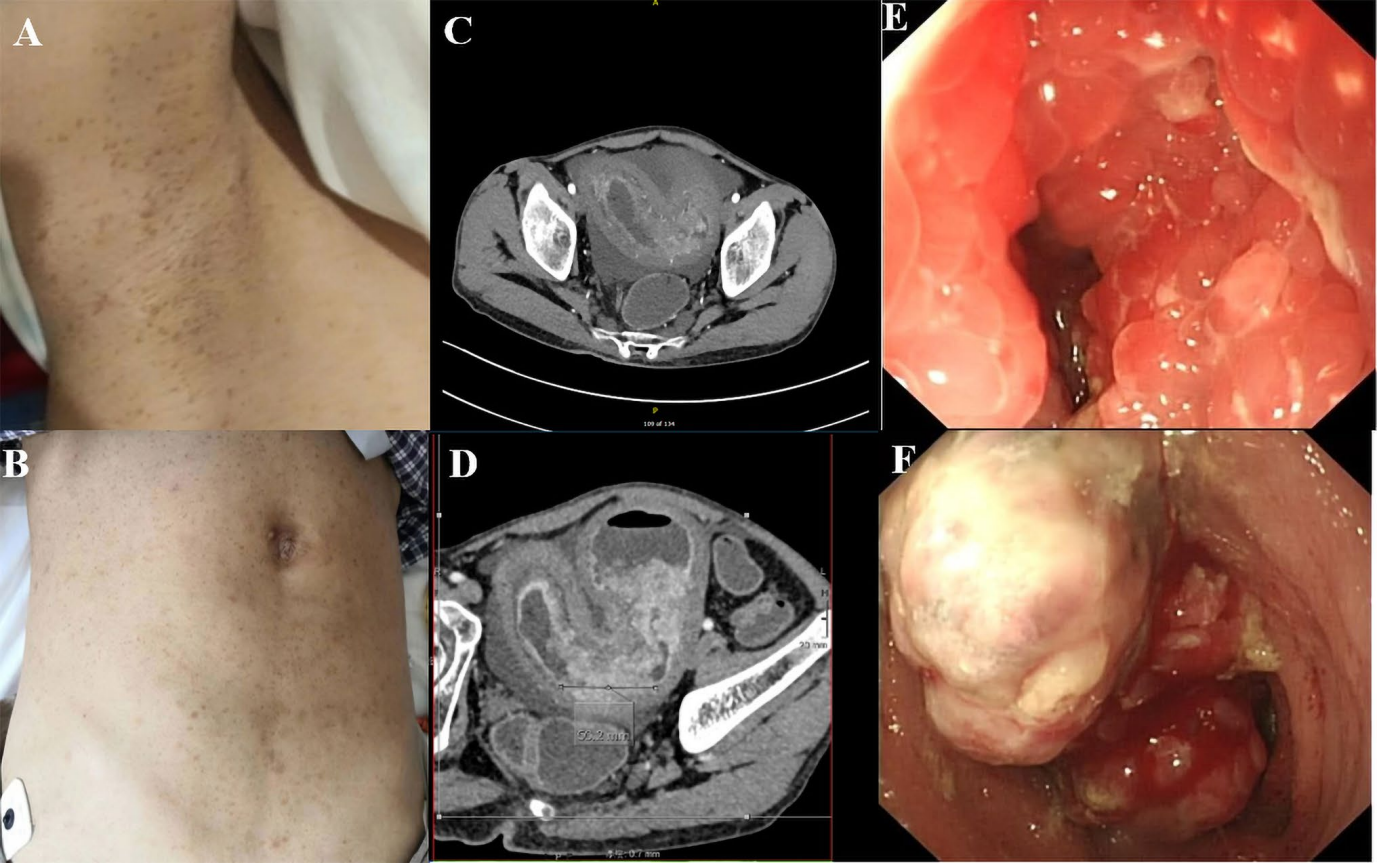
(A, B) Physical examination revealed freckling at axillary and diffused café‐au‐lait macules on skin. (C, D) Abdominal computed tomography showed marked thickening and mild enhancement in the ileal mucosa and mesentery with multiple enlarged lymph nodes. (E, F) Enteroscopy showed multiple nodular bulges in the circumferential mucosa with apparent stenosis in the lower ileum and multiple nodular protrusions covering about half of the lumen in the distal ileum.

Abdominal enhanced computed tomography (CT) showed marked thickening and mild enhancement in the ileal mucosa and mesentery with multiple enlarged lymph nodes (Figure 1C,D).

Enteroscopy showed multiple nodular bulges with apparent stenosis in the distal ileum, where the biopsy revealed inflammation (Figure 1E,F).

Laparotomy exploration revealed a 5 × 5 cm mass in the distal ileum with poor mobility and diffused mesenteric lymphadenopathy, of which the biopsy supported neurofibroma. Then, a palliative ileostomy was performed for symptom relief.

Peripheral blood whole‐exome sequencing showed 17q11.2 microdeletions involving the entire region of NF1 and adjacent genes (Figure 2A), atypical microdeletions supporting the diagnosis of Neurofibromatosis Type 1 [1].

**FIGURE 2:**
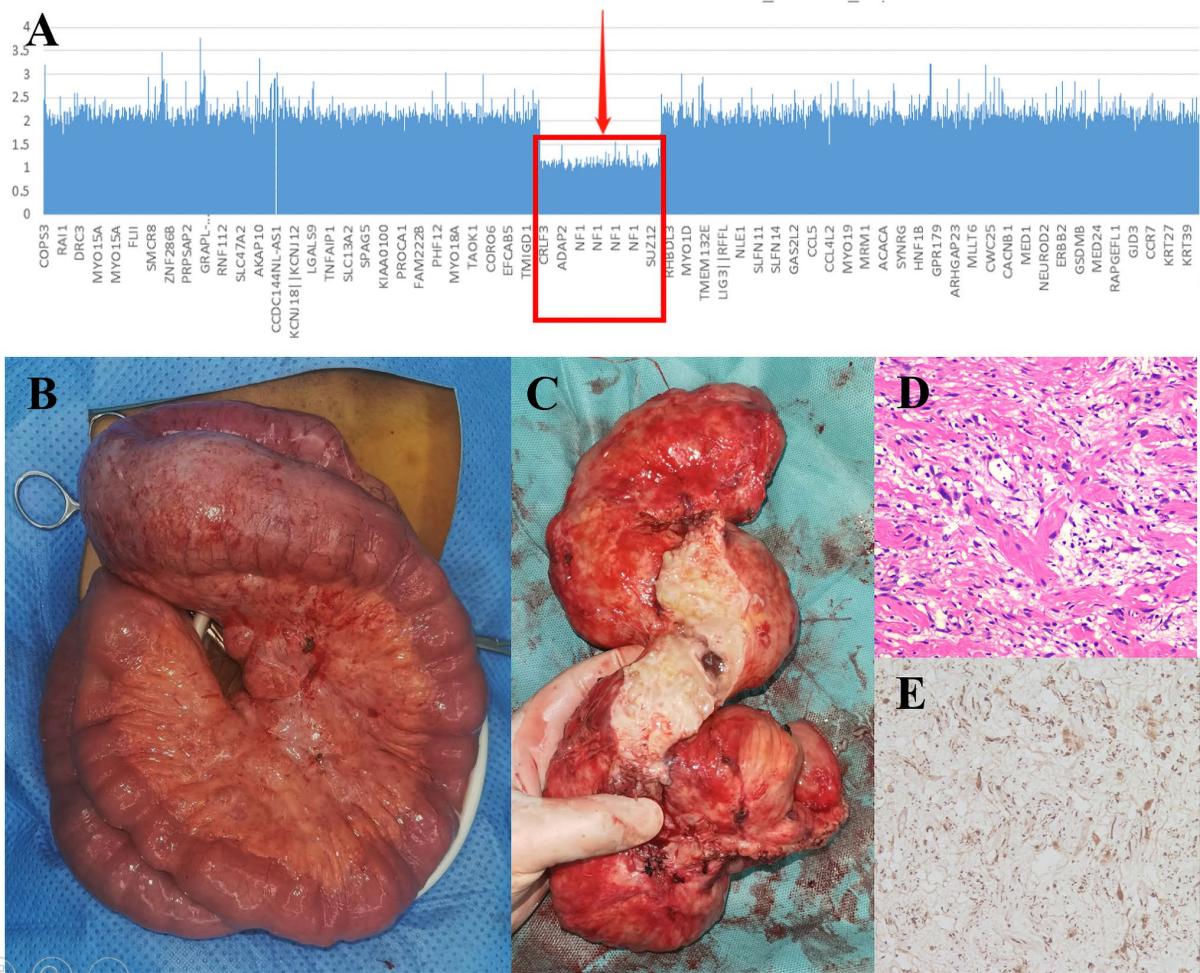
(A) Peripheral blood whole‐exome sequencing showed 17q11.2 microdeletions which include the entire NF1 and adjacent SUZ12, CRLF3, and ADAP2 genes. (B) Resected small intestine. (C) The intestinal mucosa of the small intestine cut on the longitudinal axis showed multiple polypoid protrusions. (D) Histopathological examination revealed neurofibroma. (E) Immunohistochemistry revealed positivity for S‐100.

Six months later under better self‐conditions, he underwent partial small intestine resection (Figure 2B,C) and ileostomy closure, finally achieving curable excision and functional recovery. The postoperation pathology confirmed the final diagnosis: small intestinal neurofibroma in Neurofibromatosis Type 1 (Figure 2D,E).

Neurofibromatosis Type 1 is a rare hereditary disease, of which only 5% to 11% of patients are caused by the 17q11.2 microdeletions with a more severe phenotype and complications [2]. Currently, the clinical diagnostic criteria for Neurofibromatosis Type 1 are mainly based on the consensus established by the National Institutes of Health (NIH) in 1987 (Table 1). Patients are diagnosed with Neurofibromatosis Type 1 if they have two or more of these features [3]. Our patient met the criteria of 1 and 3, and could be clearly diagnosed with Neurofibromatosis Type 1. Gastrointestinal involvement is present in 10%–25% of Neurofibromatosis Type 1 patients, but less than 5% of patients develop symptoms [4]. Small intestinal neurofibroma is even rarer since the majority of Neurofibromatosis Type 1 patients with intestinal involvement exhibit stromal tumors [5]. Neurofibromas of digestive organs in Neurofibromatosis Type 1 are very scarce, with only a few cases described in the literature (Table 2) [6, 7, 8, 9, 10, 11, 12, 13, 14, 15]. Literature review reveals that the affected sites included stomach, small intestine, mesentery, cecum, appendix, and liver. Abdominal pain is the most common symptom, often combined with gastrointestinal bleeding, perforation, obstruction, and appendiceal diverticula. No patient has gene mutations or deletions. Only one patient is found to have a diffuse mesenteric lesion that could not be completely resected for conservative treatment during surgery, and the treatment method of one patient is unknown while surgical resection is performed in the remaining cases. All cases are diagnosed as neurofibromas on histopathological examination and are benign. For neurofibromas of digestive organs in Neurofibromatosis Type 1, clinical and endoscopic manifestations lack specificity, while treatments remain controversial. Indications for surgery are not clearly defined and are mostly presented in the form of case reports. Surgical resection is seldom needed in most cases as there is limited malignant transformation potential. However, when there is a risk of malignant transformation, obstruction, and bleeding, surgical resection of the neurofibroma is required. According to the size and location of the tumor, local resection of the tumor or partial resection of the involved bowel and intestinal anastomosis are performed. This case expands the spectrum of initial clinical manifestations of Neurofibromatosis Type 1. The possibility of gastrointestinal neurofibroma in Neurofibromatosis Type 1 patients with recurrent abdominal distension should be considered. Physical examination, endoscopy, and radiographic images provide valuable visual information to other clinicians, which contributes to identifying Neurofibromatosis Type 1 at an early stage. Pathological examination and gene sequencing are critical in diagnosis, and surgical treatment should be considered when necessary.

**TABLE 1:** National Institutes of Health diagnostic criteria for Neurofibromatosis Type 1.

	Diagnostic criteria
1	Six or more café‐au‐lait macules equal to or greater than 5 mm in longest diameter in prepubertal patients and 15 mm in longest diameter in postpubertal patients
2	Two or more neurofibromas of any type or one plexiform neurofibroma
3	Freckling in the axillary or inguinal regions
4	Optic pathway glioma
5	Two or more iris hamartomas (Lisch nodules)
6	A distinctive osseous lesion, such as sphenoid wing dysplasia or long‐bone dysplasia (with associated cortical thickening and medullary canal narrowing), with or without pseudoarthrosis
7	A first‐degree relative with Neurofibromatosis Type 1

**TABLE 2:** Review of neurofibromas of digestive organs in Neurofibromatosis Type 1.

Author	Year	Sex	Age	Nation	Location	Symptom	Treatment	Complication	Mutations/deletions	Malignant transformation
Koşucu et al. [6]	2003	F	60	Turkey	Mesentery	Abdominal pain	Surgical resection	None	None	Yes
Bakker et al. [7]	2005	M	21	America	Stomach	Abdominal pain, nausea, vomiting, anorexia	Billroth II gastrojejunostomy	Gastric ulcer, gastrointestinal bleeding, gastrointestinal perforation, pyloric obstruction	None	None
Rastogi [8]	2008	M	35	India	Liver, peripancreatic region	Abdominal pain	UK	None	None	None
Rastogi [8]	2008	M	43	India	Stomach	Abdominal distention, Vomiting	Billroth II gastrojejunostomy	Pyloric obstruction	None	None
Donk et al. [9]	2011	F	69	The Netherlands	Cecum	Abdominal pain, dysphagia, anorexia	Right‐sided hemicolectomy	Appendectomy, laparoscopic cholecystectomy	None	None
Guo et al. [10]	2014	F	62	China	Appendix	Abdominal pain, abdominal distention	Surgical resection, right‐sided hemicolectomy	None	None	None
Ozaki et al. [11]	2015	M	51	Japan	Appendix	Abdominal pain	Appendectomy	Perforated appendiceal diverticulitis	None	None
Van de Steen et al. [12]	2020	M	74	Netherlands	Appendix	Abdominal pain, vomiting	Appendectomy	Appendiceal diverticula	None	None
Stević et al. [13]	2020	M	69	Serbia	Small intestinal	Anal bleeding, short‐term loss of consciousness	Surgical resection	Sigmoid colon polyps, anemia	None	None
Greenberg et al. [14]	2021	F	52	America	Appendix	Abdominal pain	Appendectomy	Abdominal infections from ventriculoperitoneal shunt	None	None
Sang et al. [15]	2022	M	5	Vietnam	Mesentery	Abdominal pain, Abdominal distention	Conservative treatment	None	None	None

## Ethics Statement

The authors are accountable for all aspects of the work in ensuring that questions related to the accuracy or integrity of any part of the work are appropriately investigated and resolved. Written informed consent was obtained from the patient for publication of this “Education and Imaging – Gastroenterology.”

## Conflicts of Interest

The authors declare no conflicts of interest.
